# Clinical Pharmacokinetics of Daprodustat: Results of an Absorption, Distribution, and Excretion Study With Intravenous Microtracer and Concomitant Oral Doses for Bioavailability Determination

**DOI:** 10.1002/cpdd.1029

**Published:** 2021-10-28

**Authors:** Kelly M. Mahar, Stephen Caltabiano, Susan Andrews, Bandi Ramanjineyulu, Liangfu Chen, Graeme Young, Adrian Pereira, Alistair C. Lindsay, Frans van den Berg, Alexander R. Cobitz

**Affiliations:** ^1^ Clinical Pharmacology Modeling & Simulation GlaxoSmithKline Collegeville Pennsylvania USA; ^2^ Development‐Clinical Sciences GlaxoSmithKline Collegeville Pennsylvania USA; ^3^ Present address: CSL Behring King of Prussia, Pennsylvania USA; ^4^ Clinical Science & Study Operations GlaxoSmithKline Research Triangle Park North Carolina USA; ^5^ Clinical Statistics GlaxoSmithKline Bangalore India; ^6^ Drug Metabolism and Pharmacokinetics GlaxoSmithKline Collegeville Pennsylvania USA; ^7^ Disposition & Biotransformation GlaxoSmithKline Ware UK; ^8^ Hammersmith Medicines Research London UK

**Keywords:** anemia, chronic kidney disease, daprodustat, hypoxia‐inducible factor, mass balance

## Abstract

Daprodustat, an oral hypoxia‐inducible factor prolyl hydroxylase inhibitor, is being investigated for treatment of anemia in chronic kidney disease. This phase 1, nonrandomized, 2‐period, crossover study in 6 healthy men characterized the absorption, distribution, and excretion of daprodustat when administered as oral and intravenous (IV) doses of unlabeled and radiolabeled daprodustat ([^14^C]‐GSK1278863). Tolerability and pharmacokinetic properties of daprodustat, and its 6 metabolites in the systemic circulation, were also evaluated. The mean recovery of radiolabeled daprodustat was ≈95% by day 5, with the majority in feces and minor renal elimination, indicating that daprodustat and metabolites are primarily eliminated via hepatobiliary and fecal routes. Approximately 40% of total circulating radioactivity in plasma following both IV and oral administration was daprodustat; thus, 60% was attributed to metabolites. It was estimated that ≈80% of daprodustat was absorbed across the gastrointestinal tract, and ≈18% cleared by hepatic extraction. Pharmacokinetics were essentially dose proportional, with moderate (≈66%) oral tablet bioavailability. Following IV administration, daprodustat plasma clearance (19.3 L/h) and volume of distribution (14.6 L) were low, suggesting low tissue distribution outside systemic circulation with likely low penetration into tissues. Daprodustat was generally well tolerated, with no deaths or serious or significant adverse events reported.

Daprodustat (GSK1278863) is an orally bioavailable hypoxia‐inducible factor (HIF) prolyl hydroxylase inhibitor (PHI) currently in phase 3 clinical studies.^1^, ^2^, ^3^ HIF‐PHIs are an emerging new class of agents that stimulate erythropoiesis through the inhibition of HIF‐prolyl hydroxylase enzymes (PHD1, PHD2, and PHD3), and are being investigated for the treatment of anemia in chronic kidney disease (CKD).^4^, ^5^ This inhibitory activity results in the accumulation of HIFα transcription factors, leading to increased transcription of HIF‐responsive genes, thereby stimulating components of the natural response to hypoxia such as erythropoietin and others involved in increasing oxygen availability and utilization. Other functions regulated by HIFs include iron metabolism and usage, apoptosis, vascular tone, angiogenesis, extracellular matrix metabolism, energy and glucose metabolism, cell adhesion, and motility.^6^

Results of in vitro studies suggest that cytochrome P450 (CYP) 2C8 is the primary CYP enzyme involved in biotransformation, with a minor contribution of CYP3A4.^7^ A clinical drug‐drug interaction study resulted in an 18.6‐fold increase in the plasma exposure of daprodustat when coadministered with gemfibrozil, likely through inhibition of CYP2C8‐mediated biotransformation. Preliminary information has been obtained on circulating metabolites following administration of daprodustat to human subjects: Components detected included unchanged daprodustat and multiple products of mono‐, di‐, and tri‐oxygenation. Six of the human circulating metabolites (GSK2391220 [M2], GSK2506104 [M3], GSK2487818 [M4], GSK2506102 [M5], GSK2531398 [M6], and GSK2531401 [M13]; structures previously reported)^7^, ^8^ are considered predominant, with higher concentrations detected in comparison to other identified metabolites.^7^ Additionally, each of these polyoxygenated metabolites exists in different positional and stereoisomeric forms, resulting in 14 separate species (2 for M13, 1 for M2, 2 for M3, 2 for M4, 3 for M5, and 4 for M6).^8^ Of the 6 metabolites, M2, M3, and M13 are considered major, as each was found to represent at least 10% of drug‐related material.^9^, ^10^ It has previously been proposed that the inhibitory potency against HIF‐prolyl hydroxylase enzymes, and selectivity against collagen prolyl hydroxylase and factor‐inhibiting HIF is similar among the predominant metabolites and daprodustat. Further, repeat dose toxicity studies in mice, rats, dogs, and monkeys suggest similar toxicologic profiles compared with daprodustat.^11^

Reported here are the results from a phase 1 study in healthy male participants that characterized daprodustat oral bioavailability, absorption, distribution, and routes and rates of excretion when administered as either single oral (solution and tablet formulations) or intravenous (IV) doses. The oral solution and IV doses of daprodustat were radiolabeled (given as [^14^C]‐GSK1278863). Additionally, the pharmacokinetic (PK) properties of daprodustat and its 6 metabolites were determined. Finally, the tolerability of daprodustat was evaluated.

## Methods

### Study Design and End Points

The primary objective of this phase 1 study was 2‐fold: (1) To determine the total radioactivity (drug‐related material) in blood and plasma following a single IV microtracer dose of [^14^C]‐GSK1278863 (concomitant with an oral tablet dose of nonradiolabeled daprodustat; period 1) and a single oral solution dose of [^14^C]‐GSK1278863 (period 2); and (2) to determine the rate and extent of excretion of total radioactivity in urine and feces and the total recovery of radioactivity following a single oral solution dose of [^14^C]‐GSK1278863. Additionally, daprodustat and metabolite concentrations in plasma following IV and oral doses of daprodustat were used to determine select PK parameters. The data from the concomitant administration of the IV microtracer and oral tablet were used to estimate daprodustat absolute bioavailability. The generated samples were also used to comprehensively characterize the biotransformation (presence and abundance of metabolites in plasma, urine, feces, and duodenal bile) of daprodustat in humans, although this was conducted separately and will be the focus of a separate publication.

This was an open‐label, single‐center, nonrandomized, 2‐period, single‐sequence, crossover, mass balance study in 6 healthy male participants (study NCT03239522 on ClinicalTrials.gov registry), conducted in accordance with Good Clinical Practice guidelines and the 2008 Declaration of Helsinki, and approved by an independent ethics committee from the Office for Research Ethics Committees Northern Ireland HSC REC A (Lisburn, BT28 2RF). This study was conducted at Hammersmith Medicines Research (London, UK). Written informed consent was obtained from each participant before enrollment.

### Daprodustat Dose

The dose of [^14^C]‐GSK1278863 administered intravenously was a microdose of 50 μg, infused over 1 hour. A microdose was selected because daprodustat had never been previously administered by IV infusion to humans. The 50 μg dose level met the criterion for a microdose for the following reasons:

- ≤100 μg
- ≤1/100th of the lowest pharmacologically active oral dose in healthy participants with normal renal function based on an increase in plasma erythropoietin^12^
- ≤1/100th of the no observed adverse effect level of 3 mg/kg/d in the 39‐week study in monkeys where gastric erosions were observed at doses of ≥10 mg/kg/d^13^

The 50‐μg total dose of [^14^C]‐GSK1278863 was formulated as a 5‐μg/mL solution in 0.9% phosphate‐buffered saline (pH adjusted between 7 and 8, with a target of 7.5; 10 mL total volume administered).

To ensure clinically relevant systemic exposure during the microdose, the IV infusion of [^14^C]‐GSK1278863 was administered concomitant with an oral nonradiolabeled 6 mg tablet dose. The oral nonradiolabeled daprodustat dose was within the therapeutic range (2‐24 mg) for the treatment of anemia due to CKD being studied in phase 3 trials and was one of the most frequently administered doses to dialysis‐dependent patients with CKD. The oral solution dose of [^14^C]‐GSK1278863 was 25 mg, which approximates the highest dose (24 mg) in the phase 3 single‐dose global studies and is approximately one‐half of the highest 3‐times‐weekly dose (48 mg) currently being investigated in a phase 3 trial. Additionally, this dose would facilitate quantitative levels of metabolites needed for the ADME end points and metabolite profiling. The [^14^C]‐GSK1278863 oral dose solution was formulated as a 200 μg/mL solution in a phosphate‐buffered saline vehicle (pH 7.5), 125 mL total volume administered.

In summary, the doses selected were suitable to meet the objectives of the study while minimizing exposure of participants to daprodustat and [^14^C]‐GSK1278863.

### Study Population

Participants were healthy men between 30 and 55 years of age (both inclusive), nonanemic (ie, hemoglobin levels at screening were greater than the lower limit of the reference range for the testing laboratory) with hemoglobin levels ≤16.0 g/dL, and body weight ≥50 kg and body mass index (BMI) within the range of 19.0 to 31 kg/m^2^ (inclusive).

Exclusion criteria included any clinically relevant abnormality identified at the screening medical assessment (physical examination/medical history), clinical laboratory tests, or 12‐lead electrocardiogram (ECG); coadministration of drugs that are inhibitors of CYP2C8 enzyme^7^; having an occupation that required monitoring for radiation exposure, nuclear medicine procedures, or excessive X‐ray radiation within the past 12 months, or received a total body radiation dose >10.0 mSv (upper limit of World Health Organization category II), or exposure to significant radiation (eg, serial X‐ray or computed tomography scans, barium meal, etc) in the 3 years before this study, or have participated in any clinical trial with exposure to ^14^C‐labeled compounds within 12 months before their first dose in this study.

### Interventions

**Figure 1:**
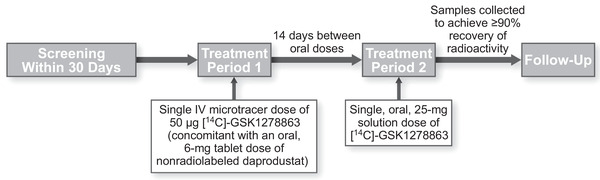
Study treatment schematic.

Each participant had a screening visit, 2 treatment periods (treatment periods 1 and 2), separated by at least 14 days between oral doses, and a follow‐up visit 1 to 2 weeks after the last assessment in treatment period 2. During both treatment periods, participants resided in the unit from the afternoon before day 1 (day −1) until all procedures were completed on day 7. Blood was sampled extensively on the day of dosing, every 12 hours on days 2 and 3, and every 24 hours thereafter until day 7, for assessment of the PK of daprodustat and total radioactive drug‐related material, as well as the 6 predominant metabolites (GSK2391220 [M2], GSK2506104 [M3], GSK2487818 [M4], GSK2506102 [M5], GSK2531398 [M6], and GSK2531401 [M13]) (Figure 1). Additionally, urine, feces, and bile samples (via Entero‐Test bile string, HDC Corp., Milpitas, California) were collected for analysis of daprodustat and its metabolites.^14^

### Treatment Period 1 (Oral Tablet and IV Infusion)

On day 1 of treatment period 1, after an overnight fast of at least 8 hours, each participant took a single 6 mg oral dose of daprodustat; participants continued to fast for < 3 hours when a small amount of food was administered to stimulate bile release for the Entero‐Test. After ≈1 hour, participants received 50 μg of [^14^C]‐GSK1278863 (≈4.63 kBq; 125 nCi) by IV infusion over 1 hour. Blood samples were collected for 144 hours after oral dosing (until day 7). Participants were discharged on study day 7 after completion of the 144‐hour sample collection. The Entero‐Test was swallowed 3.5 hours before the oral dose of daprodustat (while in the fasted state) and removed 3 hours after the oral dose (about 1 hour after completion of the IV infusion). About 1.5 hours before string withdrawal, a food cue was used to stimulate gallbladder emptying.

### Treatment Period 2 (Oral Solution)

On day 1 of treatment period 2, after an overnight fast of at least 8 hours, each participant received 25 mg [^14^C]‐GSK1278863 (≈2.31 MBq; 62.5 μCi) as an oral solution; participants continued to fast for 4 hours after dosing.^15^ Blood, urine, and fecal samples were collected for a minimum of 144 hours (up to day 7) after dosing, depending on the amount of radioactivity excreted by each participant in excreta collected daily. Liquid scintillation counting (LSC) was performed on these excreta collections on an ongoing basis to assess recovery of the radioactivity dosed. Participants were discharged if ≥90% and <1% of the dose was excreted on both days 6 and 7.

### Sample Size

As this study was descriptive in nature, no formal statistical comparisons of PK data were performed. A sample size of 6 participants, typical for this type of study, was deemed sufficient to achieve the primary objectives.^16^

#### Urine and Fecal Sample Collection and Analysis

Urine samples for measurement of total radioactivity excreted in urine were collected before dosing in period 1, then over the time periods 0 to 24, 24 to 48, 48 to 72, 72 to 96, 96 to 120, and 120 to 144 hours in period 2. An aliquot of each 24‐hour urine collection was shipped at ambient temperature to Covance (Harrogate, North Yorkshire, UK) for total radioactivity measurement by LSC. Fecal samples were collected up to 48 hours before dosing and over the same time periods as urine collection in period 2. Samples were homogenized in an appropriate volume of water: acetonitrile (ca 50:50 v/v). Total fecal samples were frozen at −70°C, then shipped on cold gel packs to Covance for total radioactivity measurement by LSC (following homogenization). Aliquots of both urine and fecal homogenates were also sent to GlaxoSmithKline (GSK) for characterization and quantification of metabolites (data not reported).

The following parameters were determined from the urine and fecal radiolabeled drug‐related material (total radioactivity) data:

- Absolute amount excreted and percentage excreted in urine (Ae [urine] and Fe% [urine]) within each collection period and cumulative urinary recovery and fraction excreted over the total collection period
- Absolute amount excreted and percentage excreted in feces (Ae [fecal] and Fe% [fecal]) with each collection period and cumulative fecal recovery and fraction excreted over the total collection period and cumulatively over the collection period

Total excretion (sum of urine and fecal excretion), Ae [total], and Fe% [total] were calculated by collection interval for each participant.

#### Blood Sample Collection and Analysis

As per standard practice for administration of radiolabeled drug, the IV and oral dosing solutions were analyzed for daprodustat via assessment of the radioactivity and through application of the predetermined specific activity. Whole blood samples were collected for measurement of each of the following: blood total radioactivity in period 2 and plasma total radioactivity in period 1, plasma concentrations of daprodustat, and the 6 predominant circulating metabolites in periods 1 and 2. Blood samples were collected at 1, 3, and 6 hours after dosing for blood assessments. Blood samples were collected for plasma assessments before dosing and at 0.50, 1, 1.25, 1.5, 2, 3, 4, 6, 8, 10, 12, 24, 36, 48, 72, 96, 120, and 144 hours after dosing.

Daprodustat and [^14^C]‐GSK1278863 plasma concentrations were measured in treatment period 1: 6 mg daprodustat oral tablet, followed by a 1‐hour IV infusion of 50 μg [^14^C]‐GSK1278863, started 1 hour after tablet administration, and in treatment period 2: 25 mg [^14^C]‐GSK1278863 oral solution.

Whole blood samples for unlabeled assays were collected into K_3_ ethylenediaminetetraacetic acid tubes, gently inverted several times and immediately placed on ice water. Samples were then centrifuged at 1500 × *g* for 15 minutes; the supernatant plasma was transferred to Nunc tubes and stored at −20°C before shipment. Samples for unlabeled daprodustat and specific assay metabolite measurements were shipped frozen to PPD Laboratories (Middleton, Wisconsin). Samples for radioactivity measurements were shipped frozen to either GSK Research and Development (Ware, Hertfordshire, UK; period 1) or Covance (period 2). Some period 2 plasma samples were subsequently analyzed by accelerator mass spectrometry (AMS) at Ware (UK) after being not quantifiable by LSC analysis.

Plasma samples from periods 1 and 2 were analyzed for daprodustat and its 6 metabolites by PPD Laboratories using a validated analytical method based on protein precipitation, followed by high‐performance liquid chromatography with tandem mass spectrometric detection analysis, as described previously.^7^, ^8^ Specifically, the lower limit of quantification (LLQ) for this study was 0.100 ng/mL using a 100 μL aliquot of ethylenediaminetetraacetic acid plasma for all 7 analytes. The higher limit of quantification was 100 ng/mL for all 7 analytes. The data accuracy (% bias range: −2.21 to −0.400 to 0.560‐5.85) and precision (% coefficient of variation of 3.99‐7.13) met acceptable limits as recommended by the US Food and Drug Administration in their bioanalytical method validation guidelines.^17^ Final PK concentration data for period 1 after 1‐hour samples for daprodustat were further corrected to account for radiolabeled ^14^C dose.

Plasma samples collected from period 1 were analyzed for [^14^C]‐GSK1278863 by GSK using a validated analytical method, based on protein precipitation, followed by liquid chromatography–AMS analysis. The LLQ was 1.80 pg/mL, using a 600 μL aliquot of human plasma with a higher limit of quantification of 358.92 pg/mL. Accuracy for the 5 validation levels were between −9.2% and 2.2%, and precision was between 2.5% and 10.8%.

Human urine, feces, and blood samples from treatment period 2 were analyzed by Covance using LSC. The LLQ for urine using LSC was 5.63 ng Eq/mL, using a 1000 μL aliquot of human urine. The LLQ for blood using LSC was 11.6 ng Eq/mL, using a 400 μL aliquot of human blood. The LLQ for feces using LSC was 25.3 ng Eq/mL, using a 0.2‐ to 0.4‐g aliquot of human feces. Treatment period 2 plasma samples that were not quantifiable following LSC analysis at Covance were subsequently reanalyzed for total radioactivity by AMS at GSK. The LLQ using AMS was 15.8 pg Eq/mL, using a 50 μL aliquot of human plasma.

### Pharmacokinetic Analysis

Plasma daprodustat and [^14^C]‐GSK1278863 plasma and blood total radioactivity concentration‐time data were analyzed by noncompartmental methods with WinNonlin Version 6.3 or above (Certara, Princeton, New Jersey). Calculations were based on the actual sampling times recorded during the study. From the plasma concentration‐time data, the following PK parameters were determined for daprodustat, its metabolites, [^14^C]‐GSK1278863, and total radioactivity, as the data permitted: maximum observed plasma concentration (C_max_), time to C_max_ (t_max_), area under the plasma concentration–time curve (AUC) from time 0 to the last measurable concentration (AUC_0‐t_), AUC from time 0 extrapolated to infinity (AUC_0–inf_), and apparent terminal phase half‐life (t_½_), following oral and IV dosing. Additionally, daprodustat volume of distribution at steady state (V_ss_) and the total systemic clearance (CL) were derived following IV dosing, and daprodustat absolute bioavailability (F) was determined from the oral tablet and IV plasma PK data from treatment period 1 with the following equation:

$$F\;=\frac{\mathrm{AU}C_{\mathrm{po}}}{\mathrm{AU}C_{\mathrm{IV}}}\;\times \frac{\mathrm{dos}e_{\mathrm{IV}}}{\mathrm{dos}e_{\mathrm{po}}}$$

To assess relative exposure levels between parent daprodustat and the measured metabolites, the ratio of parent daprodustat/total radioactivity was calculated for the plasma PK parameters following IV and oral administration of [^14^C]‐GSK1278863: C_max_, AUC_0‐t_, and AUC_0‐inf._

### Metabolite Load, Hepatic Excretion, Fraction Escaping Gut Metabolism, and Fraction Absorption Analyses

To assess the extent of metabolism and absorption of daprodustat, the plasma PK parameters: metabolite load, fraction escaping metabolism by the gut wall (F_g_), hepatic extraction (E_h_), and fraction absorbed (F_abs_) were calculated on the basis of equations found in Harrell et al.^18^ All parameters for analysis have been conducted on plasma results (assuming a blood to plasma ratio of 1) but when appropriate, the actual human blood:plasma ratio of 1.23 (internal GSK reports) of daprodustat has been applied to specific parameters to aid interpretation.

(1) $$\begin{array}{ccc} & & \mathrm{Hepatic}\;\mathrm{Extraction}:\;E_{h}\left(blood\right)\\ & & \;=\frac{\left(CL_{b\;\mathrm{total}}-CL_{b\;\mathrm{renal}}\right)}{Q_{H}} \end{array}$$

(2) $$\begin{array}{ccc} & & \text{Fraction}\;\text{escaping}\;\text{liver}\;\text{metabolism}:\\ & & \;F_{h}=\left(1-\right.\;E_{h}\;\left.\left(\mathrm{blood}\right)\right) \end{array}$$

(3) $$\begin{array}{ccc} & & \mathrm{Metabolite}\;\mathrm{Load}\left(\mathrm{TRA}=\mathrm{total}\;\mathrm{radioactivity}\right):\\ & & \;ML_{\mathrm{route}}=\;\frac{\left(\mathrm{AU}C_{\mathrm{TRA}\;\mathrm{route}}-\mathrm{AU}C_{\mathrm{route}}\right)}{\mathrm{AU}C_{\mathrm{TRA}\;\mathrm{route}}} \end{array}$$

(4) $$\text{Fraction}\;\text{escaping}\;\text{gut}:\;F_{g}=\frac{\left(1-\left(ML_{\mathrm{oral}}-ML_{\mathrm{IV}}\right)\right)}{F_{h}}$$

(5) $$\text{Fraction}\;\text{absorbed}:\;F_{\mathrm{abs}}=\frac{F_{\mathrm{oral}}}{\left(F_{h}\times F_{g}\right)}$$

When ML_IV_ is comparable (within variability) to ML_oral_, and therefore, F_g_ approaches the physiological upper limit (ie, 100%), the fraction absorbed equation can be reduced to:

(6) $$F_{abs}=\frac{F_{oral}}{\left(F_{h}\right)}\;$$

### Safety and Tolerability Measures

Safety and tolerability measures included assessment of adverse events (AEs), serious adverse events (SAEs), clinical laboratory findings, vital signs (systolic and diastolic blood pressure and pulse rate), ECGs, and concurrent medications. An AE was defined as any untoward medical occurrence in a participant temporally associated with the use of an investigational product, whether or not considered related to the investigational product. An SAE was defined as any untoward medical occurrence that, at any dose, resulted in death, was life threatening, required hospitalization or prolonged existing hospitalization, resulted in disability/incapacity, was a congenital anomaly/birth defect, or was associated with liver injury and impaired liver function. The investigator or site staff was responsible for detecting, documenting, and reporting events that met the definition of an AE or SAE.

## Results

### Participant Population

A total of 6 healthy adult male participants were enrolled in this study, with all enrolled participants completing both treatment periods and study assessments as planned. The mean age and BMI were 40.7 years and 23.52 kg/m^2^, respectively. This study was conducted between October 10, 2017, and November 29, 2017. Demographic characteristics of the study population can be found in Table 1.

**Table 1:** Demographic Characteristics of the Study Population

Parameter (Units)	Mean ± SD^a^
Age, y	40.7 ± 8.57
Height, cm	181.2 ± 4.49
Weight, kg	77.03 ± 8.437
BMI, kg/m^2^	23.52 ± 2.892

BMI, body mass index; N, number of participants in the full PK population; SD, standard deviation.

^a^ N = 6 participants completed treatment periods 1 and 2.

### Urine and Fecal Excretion

**Figure 2:**
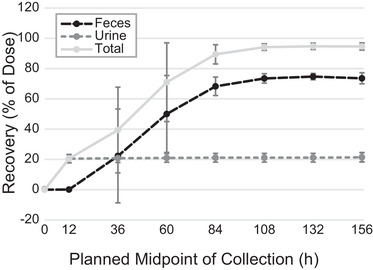
Cumulative radioactivity recovered from urine, feces, and total excretion following 25 mg of an oral daprodustat solution. Bars represent arithmetic mean ± standard deviation as a percentage of administered dose.

Cumulative urinary and fecal excretion of radioactivity following administration of 25 mg [^14^C]‐GSK1278863 (oral solution; treatment B, period 2) as a percentage of the administered radioactivity is shown in Figure 2. Urinary radioactivity excreted remained consistent at ≈20% of the administered radioactivity (unchanged daprodustat accounted for ≈0.5%), while fecal excretion increased with time and remained relatively constant at ≈74% starting at the 96‐ to 120‐hour collection period. Total radioactivity (ie, urinary + fecal) reached ≈95% during the 96‐ to 120‐hour collection period and remained relatively constant until the end of the radioactive collection (144‐196 hours). Daprodustat renal clearance was not measured in this study, as this was reported for healthy volunteers in an earlier determination (≈0.20 mL/min).^10^

### Daprodustat PK

**Figure 3:**
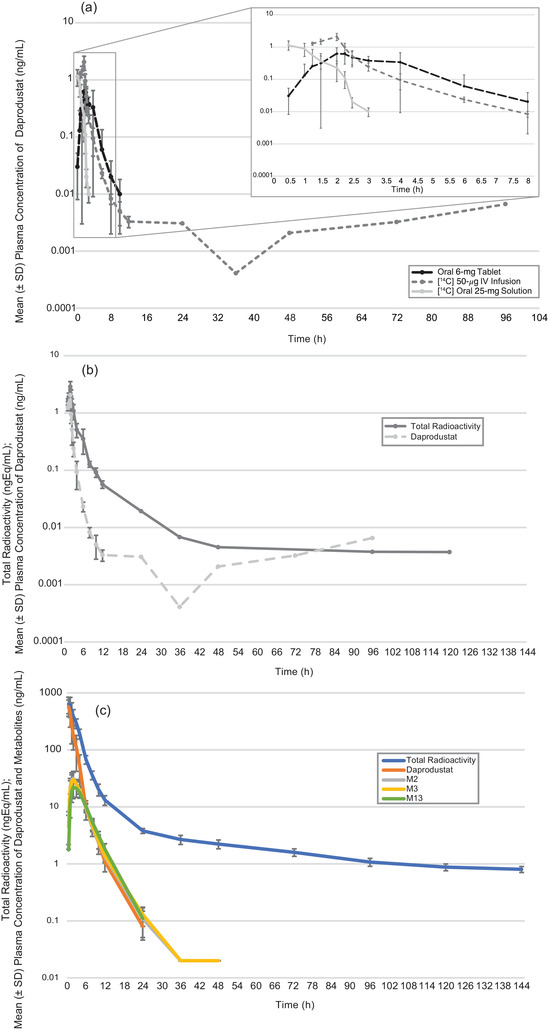
Arithmetic mean ± standard deviation (SD) concentrations of (a) plasma daprodustat following IV administration with concomitant oral tablet administration and oral solution administration; inset plot is *x*‐axis truncated to 8 hours (oral tablet and solution dose concentration data are normalized to the 50 μg IV dose); (b) total radioactivity and daprodustat in plasma following IV administration; and (c) total radioactivity, daprodustat, and major metabolites M2, M3, and M13 in plasma following oral solution administration.

Plasma daprodustat PK and PK parameters by treatment are summarized in Table 2 and in Figure 3. Following oral tablet administration, daprodustat plasma concentrations were quantifiable up to 12 hours after dosing in all participants, with the plasma concentrations declining rapidly immediately following C_max_ (median t_max_ ≈2 hours after dosing), and declining more slowly thereafter. Following IV administration, [^14^C]‐GSK1278863 plasma concentrations of daprodustat were quantifiable up to 10 hours after dosing in most participants. C_max_ was attained immediately following the end of the 1‐hour infusion (median t_max_ = 1 hour), and plasma [^14^C]‐GSK1278863 concentrations declined rapidly to 10 hours after dosing. Following oral solution administration of [^14^C]‐GSK1278863, daprodustat plasma concentrations peaked quickly following administration and reached C_max_ with a median t_max_ at ≈0.5 hour.

**Table 2:** Plasma Daprodustat Pharmacokinetic Parameters by Treatment

Parameter (Units)^a^	6 mg Oral Tablet	N = 6	[^14^C] 50 μg IV Infusion^b^	N = 6	[^14^C] 25 mg Oral Solution	N = 6
C_max_, ng/mL	97.3 (16.4)	n = 6	2.06 (0.53)	n = 6	570 (183)	n = 6
t_max_, h	2.12 (1.42−4.00)	n = 6	0.983 (0.983−0.983)	n = 6	0.500 (0.500−0.517)	n = 6
AUC_0‐inf_, ng • h/mL	202 (36.5)	n = 6	2.71 (0.69)	n = 3	974 (301)	n = 6
AUC_0‐t,_ ng • h/mL	201 (35.9)	n = 6	2.68 (0.53)	n = 6	972 (301)	n = 6
t_½_, h	1.92 (0.43)	n = 6	2.07 (0.13)	n = 3	2.60 (0.75)	n = 6
CL, L/h	NA	NA	19.3 (5.36)	n = 3	NA	NA
V_ss_, L	NA	NA	14.6 (3.46)	n = 3	NA	NA
Oral F (0‐inf)	0.661 (0.077)	n = 3	NA	NA	NA	NA

AUC_0‐inf,_ area under the concentration‐time curve from time 0 (predose) extrapolated to infinity; AUC_0‐t_, AUC up to the last measurable concentration; CL, clearance; C_max_, maximum observed plasma concentration; N, number of participants in the full PK population; n, number of participants with quantifiable data for parameter calculation; NA, not applicable; Oral F, oral absolute bioavailability; PK, pharmacokinetic; SD, standard deviation; t_max_, time to C_max_; t_½_, terminal phase half‐life; V_ss_, volume of distribution at steady state.

^a^ Values expressed are arithmetic mean (SD) except t_max_, which is given as median (range).

^b^ 1‐hour infusion started at 1 hour after oral dose.

Daprodustat plasma concentrations following oral administration were appreciably higher after administration of the 25 mg solution dose than the 6 mg tablet dose as expected due to the increase in dose. For the 4.17 difference in dose, the mean C_max_ and AUC_0‐t_ were 5.86‐fold and 4.84‐fold greater, respectively, for solution vs tablet. For the 120‐fold difference between the 6 mg tablet and the 50 μg IV dose, oral daprodustat mean C_max_ and AUC_0‐t_ were 47.2‐fold and 75.0‐fold greater, respectively, during treatment period 1.

Mean terminal elimination half‐lives were similar for daprodustat following oral and IV administration. Following IV administration of [^14^C]‐GSK1278863, mean CL of daprodustat from plasma was low, 19.3 L/h, which translates to a blood CL of ≈15 L/h, and using Equation 1 equates to a hepatic extraction of ≈18% (human liver blood flow of 87 L/h).^19^ The mean V_ss_ was low, 14.6 L, approximating that of extracellular fluid (18.2 L),^19^ suggesting low tissue distribution of the drug outside the systemic circulation with likely low penetration into tissues.

### Absolute Bioavailability

The absolute bioavailability (oral F) of daprodustat administered as a 6 mg oral dose was 66.1% based on AUC_0‐inf_ indicating moderate bioavailability (Table 2).

### Blood and Plasma Total Radioactivity

The shape of the plasma total radioactivity profiles following oral and IV administration were very similar to the [^14^C]‐GSK1278863 and daprodustat profiles (Figures 3B and 3C). In addition, plasma total radioactivity was detectable for longer than daprodustat plasma concentrations with quantifiable values up to 120 hours and 144 hours, following IV and oral administration, respectively. This is expected as the radioactivity bioanalytical method had a more sensitive limit of quantification (1.8 vs 100 pg/mL).

On average, the total plasma radioactivity following IV and oral administration reached maximum levels at 0.98 hour and 0.76 hour after dosing, respectively (median t_max_ estimates; Table 3). The systemic exposure as measured by AUC_0‐inf_ was relatively high following oral administration and consistent with moderate bioavailability of the orally administered drug. The mean CL estimates from plasma total radioactivity were several‐fold lower following IV administration compared to daprodustat IV CL. V_ss_ estimates from plasma total radioactivity were higher compared to daprodustat IV V_ss_.

**Table 3:** Total Radioactivity Pharmacokinetic Parameters^a^ by Specimen and Treatment

PK Parameter (Units)	[^14^C]‐GSK1278863 Treatment	N = 6	Specimen	Arithmetic Mean (SD)
C_max_, ng Eq/mL	IV infusion	n = 6	Plasma	3.04 (0.49)
	Oral solution	n = 6	Blood	302 (95.6)
	Oral solution	n = 6	Plasma	654 (219)
t_max_, h	IV infusion	n = 6	Plasma	0.983 (0.983−1.25)
	Oral solution	n = 6	Blood	1.00 (1.00−3.00)
	Oral solution	n = 6	Plasma	0.758 (0.500−3.00)
AUC_0‐inf_, ng Eq • h/mL	IV infusion	n = 5	Plasma	6.79 (1.31)
	Oral solution	n = 6	Plasma	2312 (447)
AUC_0‐t_, ng Eq • h/mL	IV infusion	n = 6	Plasma	6.62 (1.20)
	Oral solution	n = 6	Blood	835 (154)^b^
	Oral solution	n = 6	Plasma	2239 (441)
t_½_, h	IV infusion	n = 5	Plasma	8.88 (6.58)
	Oral solution	n = 6	Plasma	62.4 (9.62)
CL, μg/[ng Eq • h/mL]	IV infusion	n = 5	Plasma	7.59 (1.50)
V_ss_, μg/[ng Eq/mL]	IV infusion	n = 5	Plasma	36.3 (19.6)

AUC_0‐inf_, area under the concentration‐time curve from time 0 (predose) extrapolated to infinite time; AUC_0‐t_, AUC up to the last measurable concentration; CL, clearance; C_max_, maximum observed plasma concentration; N, number of participants in the full PK population; n, number of participants with quantifiable data for parameter calculation; PK, pharmacokinetic; t_max_, time to C_max_; SD, standard deviation; t_½_, terminal phase half‐life; V_ss_, volume of distribution at steady state.

^a^ Values expressed are arithmetic mean (SD) except t_max_, which is given as median (range).

^b^ t = 6 hours; blood PK data for treatment B were available only to 6 hours, and therefore not all PK parameters could be calculated.

### Metabolite PK

During treatment period 2, following oral solution administration, C_max_ and systemic exposure of metabolites were higher than during treatment period 1 following oral tablet administration (Table 4), and t_max_ values were attained sooner with slightly longer t_½_ estimates after solution dosing for all measured daprodustat metabolites, with a more marked increase of t_½_ observed for GSK2391220 (M2) and GSK2506104 (M3).

**Table 4:** Plasma Daprodustat Metabolite Pharmacokinetic Parameters^a^ by Treatment

Analyte	Parameter (Units)	Oral 6 mg Tablet	N = 6	[^14^C] Oral 25 mg Solution	N = 6
GSK2391220 (M2)	C_max_ (ng/mL)	7.59 (1.05)	n = 6	32.7 (9.38)	n = 6
	t_max_ (h)	3.50 (2.98−6.00)	n = 6	2.00 (1.50−4.00)	n = 6
	AUC_0‐inf_ (ng • h/mL)	34.1 (5.02)	n = 6	142 (27.0)	n = 5
	t_½_ (h)	1.99 (0.16)	n = 6	3.23 (0.71)	n = 5
GSK2506104 (M3)	C_max_ (ng/mL)	7.42 (0.96)	n = 6	31.4 (8.23)	n = 6
	t_max_ (h)	4.00 (2.98−6.00)	n = 6	2.00 (2.00−4.00)	n = 6
	AUC_0‐inf_ (ng • h/mL)	34.3 (3.95)	n = 6	142 (23.3)	n = 5
	t_½_ (h)	2.09 (0.17)	n = 6	3.40 (0.36)	n = 5
GSK2487818 (M4)	C_max_ (ng/mL)	6.13 (1.17)	n = 6	26.6 (8.30)	n = 6
	t_max_ (h)	3.50 (1.98−6.00)	n = 6	2.00 (1.50−4.00)	n = 6
	AUC_0‐inf_ (ng • h/mL)	22.0 (3.84)	n = 6	93.1 (15.8)	n = 6
	t_½_ (h)	1.58 (0.20)	n = 6	1.87 (0.20)	n = 6
GSK2506102 (M5)	C_max_ (ng/mL)	1.84 (0.29)	n = 6	7.00 (1.77)	n = 6
	t_max_ (h)	3.50 (2.98−6.00)	n = 6	2.00 (2.00−4.00)	n = 6
	AUC_0‐inf_ (ng • h/mL)	8.70 (1.29)	n = 6	33.5 (5.04)	n = 6
	t_½_ (h)	2.02 (0.32)	n = 6	2.28 (0.12)	n = 6
GSK2531398 (M6)	C_max_ (ng/mL)	3.55 (0.46)	n = 6	13.9 (3.51)	n = 6
	t_max_ (h)	3.50 (2.98−6.00)	n = 6	2.00 (2.00−4.00)	n = 6
	AUC_0‐inf_ (ng • h/mL)	15.1 (2.05)	n = 6	62.4 (9.57)	n = 6
	t_½_ (h)	1.72 (0.24)	n = 6	1.98 (0.07)	n = 6
GSK2531401 (M13)	C_max_ (ng/mL)	6.58 (1.61)	n = 6	23.0 (7.05)	n = 6
	t_max_ (h)	4.00 (3.00−6.00)	n = 6	2.00 (2.00−4.00)	n = 6
	AUC_0‐inf_ (ng • h/mL)	35.2 (7.94)	n = 6	127 (28.5)	n = 6
	t_½_ (h)	2.34 (0.29)	n = 6	2.93 (0.28)	n = 6

AUC_0‐inf_, area under the concentration‐time curve from time 0 (predose) extrapolated to infinity; AUC_0‐t_, AUC up to the last measurable concentration; C_max_, maximum observed plasma concentration; N, number of participants in the full PK population; n, number of participants with quantifiable data for parameter calculation; PK, pharmacokinetic; t_max_, time to C_max_; SD, standard deviation; t_½_, terminal phase half‐life.

^a^ Values expressed are arithmetic mean (SD) except t_max_, which is given as median (range).

C_max_, AUC_0‐inf_, and AUC_0‐t_ increased in a dose‐proportional manner for all measured daprodustat metabolites for an increase in dose from 6 mg (oral tablet) to 25 mg (oral solution).

Using the data reported in Tables 2 and 3, the arithmetic mean C_max_, AUC_0‐t_, and AUC_0‐inf_ ratios of plasma daprodustat to plasma total radioactivity were 0.68, 0.40, and 0.40, respectively, following IV administration and 0.87, 0.43, and 0.42, respectively, following oral solution administration. The AUC exposure ratios indicate that parent daprodustat accounted for ≈40% of total circulating radioactivity in the plasma following IV and oral solution administration. Thus, 60% of radioactivity may reside as daprodustat metabolites.

### Metabolite Load, Hepatic Excretion, Fraction Escaping Gut Metabolism, and Fraction Absorbed

Plasma metabolite load (ratio of metabolites to total radioactivity in the systemic circulation) following IV and oral administration was similar, suggesting limited metabolism within the gut wall. Following oral solution administration, an estimated 81% of daprodustat is absorbed across the gastrointestinal (GI) tract, and hepatic extraction was estimated at 18% (Table 5).

**Table 5:** Summary of Plasma Metabolite Load, Hepatic Extraction, and Absorption Parameters for Daprodustat

Parameter	Treatment	N = 6	Arithmetic Mean (SD)	95% Confidence Interval
ML_IV_	[^14^C] IV infusion	n = 3	0.623 (0.080)	(0.424‐0.822)
ML_PO_	[^14^C] oral solution	n = 6	0.584 (0.060)	(0.520‐0.647)
F_g_	[^14^C] oral solution	n = 3	1.27 (0.071)	(1.09‐1.44)
E_h_ ^a^	[^14^C] IV infusion	n = 3	0.181 (0.050)	(0.056‐0.305)
F_abs_ ^a^, ^b^	[^14^C] oral solution	n = 3	0.811 (0.134)	(0.477‐1.14)

E_h_, hepatic extraction; F_abs_, fraction absorbed; F_g_, fraction escaping metabolism by the gut wall; ML, metabolite load; N, number of participants in the full PK population; n, number of participants with quantifiable data for parameter calculation.

^a^ Values calculated from plasma and adjustment with blood:plasma ratio of 1.23.

^b^ F_abs_ was reduced to F_oral_/F_h_ as F_g_ approached limit of 1 due to negligible gut wall metabolism.

### Safety Results

No deaths or other SAEs were reported during this study.

A total of 7 AEs were reported in 3 participants (50%; Table 6). All AEs resolved, and none led to withdrawal from the study. There were no clinical laboratory evaluations, ECGs, or vital sign values considered to be clinically significant.

**Table 6:** Summary of Adverse Events Reported by All Participants

Preferred Term, n (%)	Period 1 (N = 6)	Period 2 (N = 6)	Total (N = 6)
Back pain	1 (17)	0	1 (17)
Musculoskeletal chest pain	0	1 (17)	1 (17)
Myalgia	1 (17)	0	1 (17)
Decreased appetite	1 (17)	0	1 (17)
Headache	1 (17)	1 (17)	1 (17)
Oropharyngeal pain	0	1 (17)	1 (17)

N, number of participants in the full PK population.

Period 1: Single 6‐mg oral daprodustat tablet + 50‐μg intravenous [^14^C]‐GSK1278863.

Period 2: Single 25‐mg oral [^14^C]‐GSK1278863 solution.

Numbers in parentheses are the % of participants reporting the adverse event.

## Discussion

The absorption, disposition, and excretion of daprodustat was characterized in 6 healthy, adult male participants. Following administration of [^14^C]‐GSK1278863 as a 25 mg oral solution, the mean total recovery of administered radiolabeled daprodustat was somewhat variable in the first 72 hours among the 6 participants but very consistently recovered between 72 and 168 hours. Approximately 95% of radioactivity was recovered, with the majority (73.6% of 95%) in the feces. Renal excretion played a minor role and accounted for ≈21.4% of the administered radiolabeled dose, with the majority occurring during the first 24 hours after dosing. This would indicate that daprodustat and metabolites are principally eliminated via the hepatobiliary and fecal routes. Qualitative characterization of daprodustat elimination in bile, as well as a comprehensive summary of the quantification and characterization of the metabolic routes for daprodustat, has been reported elsewhere.^20^

The PK of daprodustat administered via IV infusion, oral tablet, or oral solution all resulted in parameter estimates that were well characterized with low variability among the 6 healthy volunteers. C_max_ of daprodustat, administered as either a 6 mg oral tablet or 25 mg oral solution, was essentially dose proportional, with a 5.86‐fold increase in C_max_ with a 4.17‐fold increase in dose. Total exposure (AUC_0‐inf_) was slightly greater than dose proportional, with a 4.84‐fold increase. This slight deviation from dose proportionality appears to be due to both the greater relative bioavailability of the oral solution and the ability to measure plasma daprodustat concentrations out to 10 hours after dosing in most participants following the nonradiolabeled tablet, while daprodustat was typically quantifiable out to 12 hours following the oral solution due to the larger dose administered. However, the PK of daprodustat administered as a 25 mg oral solution was comparable to what has previously been observed with a 25 mg tablet dose.^21^

An IV microtracer concomitant administration with oral dosing is a well‐established approach^22^ ^−^ ^25^ to determine absolute bioavailability. A 1‐hour IV infusion of a 50 μg dose of [^14^C]‐GSK1278863 was used, with infusion initiated at 1 hour after dosing of the oral tablet, near the estimated oral t_max_ to ensure clinically relevant systemic exposure (measured t_max_, 2.12 hours), an approach that has shown good agreement with regular dosing.^26^ Under these conditions, absolute oral bioavailability as estimated was ≈66%, a value suggesting moderate bioavailability. Factors affecting oral bioavailability were not assessed in this study, but may be due to first‐pass metabolism, since it is known that daprodustat is extensively metabolized by CYP2C8.^7^

Following administration of [^14^C]‐GSK1278863 as an IV or oral solution, total radioactivity PK properties in blood and plasma were determined and found to have low variability among these 6 healthy volunteers. Considering the C_max_ following oral solution administration from blood (302 ng Eq/mL) and plasma (654 ng Eq/mL), a 0.46 blood:plasma ratio was observed. This suggests that there is a low association of daprodustat and metabolites to red blood cells, with much of the radioactivity likely in the plasma. As this is different from the daprodustat blood:plasma ratio of 1.23, it appears that the metabolites are less associated with red blood cells than parent drug despite the high level of daprodustat protein binding (>99%) that has been observed (GSK data on file). Furthermore, the V_ss_ estimates from plasma total radioactivity were higher than parent, indicating that the metabolites are more widely distributed than the parent drug, which is reflected by the lower CL value for plasma total radioactivity.

The PK properties of the 6 metabolites present in the circulation following solution administration were determined and each metabolite PK found to be very similar among the 6 healthy volunteer participants. Consistent with previous reports in healthy participants,^7^ following administration of [^14^C]‐GSK1278863 25 mg oral solution, the C_max_ values of the metabolites after a single dose were low relative to daprodustat, ranging from 7.00 ng/mL (M5) to 32.7 ng/mL (M2) (daprodustat C_max_ = 570 ng/mL). Similarly, AUC_0‐inf_ values of the metabolites in this study were also lower than daprodustat, ranging from 33.5 ng·h/mL (M5) to 142 ng·h/mL (M2 and M3) (daprodustat AUC_0‐inf_ = 974 ng·h/mL). In this study in healthy volunteers, similar t_1/2_ values to those of daprodustat following administration of both oral tablet and solution were observed for each metabolite (Table 4, Figure 3C), thus suggesting formation of rate‐limited kinetics. A detailed description of the metabolic fate of daprodustat is reported elsewhere.^20^

The plasma metabolite load (ratio of metabolite to total radioactivity) following IV and oral delivery was able to be reliably calculated for only 3 of the 6 participants due to a high last observation data point in 3 participants. The calculated metabolite load values for these participants were similar, and the means and standard deviations were ≈0.623 (0.080) and 0.584 (0.060), suggesting limited metabolism within the gut wall. This was expected as daprodustat is extensively metabolized by CYP2C8, which is not expressed at high levels in the gut.^27^ Following oral solution administration, it is estimated that ≈81% of daprodustat is absorbed (F_abs_) across the GI tract, and ≈18% of the drug is cleared (E_h_) before reaching the plasma. The individually calculated F_g_ values resulted in a mean and 95% confidence interval of values >1; although this is not physiologically possible and a mathematical artifact, F_g_ was fixed to 100%, as this is the physiological upper limit.

Daprodustat, administered as an oral tablet, microtracer IV, or oral solution dose, was generally well tolerated, and no new safety concerns were identified. There were no deaths or other SAEs during the study. The majority of the AEs were mild in intensity and none led to withdrawal from the study. There were no clinical laboratory values that were of potential clinical importance, and no ECG or vital sign value of potential clinical importance deemed an AE.

## Conclusion

Overall, daprodustat exhibited moderate oral bioavailability and rapid absorption. Approximately 40% of total circulating radioactivity (AUC ratio of daprodustat:total radioactivity) in the plasma following both IV and oral administration was daprodustat; thus, 60% of radioactivity is likely daprodustat metabolites. Fecal excretion was the major elimination pathway of total radioactivity with renal CL as a minor route of elimination, occurring principally during the first 24 hours after dosing. This would indicate that daprodustat and metabolites are primarily eliminated via the hepatobiliary and fecal routes. From the metabolite load assessment, it was estimated that ≈81% of daprodustat is absorbed across the GI tract, and ≈18% of the drug is cleared before reaching the plasma. PK properties were essentially dose proportional, with moderate (≈66%) oral absolute bioavailability for the 6 mg tablet. Following IV administration, daprodustat plasma CL was low, as was the volume of distribution, suggesting low tissue distribution of the drug outside the systemic circulation with likely low penetration into tissues. The results from this study will further contribute to the understanding of the clinical pharmacology of daprodustat.

## Conflicts of Interest

K.M.M., L.C., A.C.L., and A.R.C. are employees of and shareholders in GlaxoSmithKline (GSK). S.C. is a former employee and stockholder in GSK. S.A., B.R., G.Y., and A.P. are employees of GSK, F.v.d.B.’s institution (Hammersmith Medicines Research) was paid by GSK to run the clinical part of the trial.

## Funding

This study was funded by GlaxoSmithKline.

## Data‐Sharing Statement

Anonymized individual participant data and study documents can be requested for further research from www.clinicalstudydatarequest.com.
